# Fungal lysozyme leverages the gut microbiota to curb DSS-induced colitis

**DOI:** 10.1080/19490976.2021.1988836

**Published:** 2021-10-25

**Authors:** Ida Søgaard Larsen, Benjamin A. H. Jensen, Erica Bonazzi, Béatrice S. Y. Choi, Nanna Ny Kristensen, Esben Gjerløff Wedebye Schmidt, Annika Süenderhauf, Laurence Morin, Peter Bjarke Olsen, Lea Benedicte Skov Hansen, Torsten Schröder, Christian Sina, Benoît Chassaing, André Marette

**Affiliations:** a Quebec Heart and Lung Institute (Iucpq), Faculty of Medicine, and Institute of Nutrition and Functional Foods (INAF), Laval University, Québec, Canada; b Novo Nordisk Foundation Center for Basic Metabolic Research, Faculty of Health and Medical Sciences, University of Copenhagen, Copenhagen, Denmark; c Department of Biomedical Sciences, Faculty of Health and Medical Sciences, University of Copenhagen, Copenhagen, Denmark; d Inserm U1016, Team “Mucosal Microbiota in Chronic Inflammatory Diseases”, Université De Paris, Paris, France; e Novozymes A/S, Bagsværd, Denmark; f Institute of Nutritional Medicine, University Hospital Schleswig-Holstein, Lübeck, Germany

**Keywords:** Gut health, high fat diet, mucus, colitis, insulin resistance, muramidase, microbiota encroachment, intestinal inflammation, microbiota function, host defense peptides

## Abstract

Colitis is characterized by colonic inflammation and impaired gut health. Both features aggravate obesity and insulin resistance. Host defense peptides (HDPs) are key regulators of gut homeostasis and generally malfunctioning in above-mentioned conditions. We aimed here to improve bowel function in diet-induced obesity and chemically induced colitis through daily oral administration of lysozyme, a well-characterized HDP, derived from *Acremonium alcalophilum*.

C57BL6/J mice were fed either low-fat reference diet or HFD ± daily gavage of lysozyme for 12 weeks, followed by metabolic assessment and evaluation of colonic microbiota encroachment. To further evaluate the efficacy of intestinal inflammation, we next supplemented chow-fed BALB/c mice with lysozyme during Dextran Sulfate Sodium (DSS)-induced colitis in either conventional or microbiota-depleted mice. We assessed longitudinal microbiome alterations by 16S amplicon sequencing in both models.

Lysozyme dose-dependently alleviated intestinal inflammation in DSS-challenged mice and further protected against HFD-induced microbiota encroachment and fasting hyperinsulinemia. Observed improvements of intestinal health relied on a complex gut flora, with the observation that microbiota depletion abrogated lysozyme’s capacity to mitigate DSS-induced colitis.

*Akkermansia muciniphila* associated with impaired gut health in both models, a trajectory that was mitigated by lysozyme administration. In agreement with this notion, PICRUSt2 analysis revealed specific pathways consistently affected by lysozyme administration, independent of vivarium, disease model and mouse strain.

Taking together, lysozyme leveraged the gut microbiota to curb DSS-induced inflammation, alleviated HFD-induced gastrointestinal disturbances and lowered fasting insulin levels in obese mice. Collectively, these data present *A. alcalophilum-*derived lysozyme as a promising candidate to enhance gut health.

## Introduction

Host-microbe mutualism has emerged as a potent regulator of both gastro-intestinal (GI) and extra-intestinal diseases, including inflammatory bowel disease (IBD), obesity, and insulin resistance (IR). Overnutrition and high fat diet (HFD) consumption alter gut microbiota composition and reduce intestinal barrier function, thereby promoting low-grade inflammation.^1–6^ Gut bacteria are species-dependently affecting IR^7–9^ thus advocating a causal link between aberrant microbiota and altered metabolic health. Bacterial remnants are, as a result of intestinal barrier dysfunction, further capable of translocating to circulation, where lipopolysaccharides (LPS) from Gram-negative bacteria exacerbate IR.^10^ Host defense peptides (HDPs) patrol the intestinal barrier to keep intestinal microbes in check, hence mitigating bacterial translocation.^11^ These peptides are generally downregulated in human obesity and Type 2 Diabetes (T2D),^12,13^ potentially enabling microbiota encroachment and translocation to extra-intestinal tissues.^14^ Microbiota encroachment, i.e. a reduced distance from bacteria to the intestinal epithelial cells, correlates with impaired glycemic control.^15^ Recently, we and others reported bacterial translocation to key metabolic tissues in human obesity, where the site-specific community structures discriminated between weight-matched individuals with and without T2D.^16,17^ These findings add to the accumulating evidence of bacterial involvement in the onset and prevention of glucoregulatory impairments.

Compared to obesity, gut health is further disrupted in IBD with clear genetic links to diminished HDP production.^18,19^ Bacterial translocation is well established in patients with IBD^20,21^ associating with reduced gut barrier function^22^ and a change in gut microbiota composition.^23,24^ Thus, obesity and IBD exhibit similar GI complications, although the links between the two phenotypes remain inadequately described.^25^ When the gut microbiota was evaluated by 16S rRNA gene amplicons, no similarities in microbiota composition changes within patients with obesity and IBD were reported.^26^ However, with more advanced network analysis, common regulation of specific enzymes involved in the phosphotransferase system or the nitrate reductase pathway has been identified between these patient groups^27^ supported by a change in bacterial co-abundances.^28^ Similarly, frameshift mutations in the pattern recognition receptor, nucleotide-binding oligomerization domain-containing protein 2 (NOD2), predispose for human IBD,^29^ while genetic ablation of the same protein promotes IR in HFD-fed mice.^30^ Patients with IBD exhibit increased prevalence of IR and nonalcoholic fatty liver disease (NAFLD), thus supporting a close connection between the mentioned diseases.^31,32^ Still, IR and IBD are rarely studied together, although both pathologies are increasing worldwide.^33,34^

We have recently demonstrated beneficial traits of the two HDPs, human α defensin 5 (HD-5) and -β defensin 2 (hBD-2) on HFD-induced IR and experimental colitis, respectively.^35,36^ Lysozyme, also known as muramidase, was the first identified HDP.^37^ Lysozyme degrades peptidoglycans (PGN), thereby targeting both Gram negative and -positive bacteria, ultimately protecting the host from bacterial invasion.^11^ Enhanced bacterial translocation in human obesity^16,17^ may partly relate to decreased HDP secretion,^14^ wherein particular fecal lysozyme abundance inversely correlates with hyperglycemia.^12^ In humans, lysozyme is most abundantly produced by small intestinal Paneth cells. Paneth cell hyperplasia, where small intestinal Paneth cells ‘leaks’ to descending colon, is a well-described phenomenon following gut inflammation in IBD^38^ enhancing colonic lysozyme activity.^39^ Endogenous lysozyme therefore seems discordantly regulated and spatially separated in obesity^12^ and IBD.^39^ Curiously, Yu et al. recently reported that lysozyme-mediated degradation of hallmark IBD-associated microbes, such as *Rumunicoccus gnavus*, facilitated gut inflammation rather than protecting against disease burden,^40^ hence presenting lysozyme as a potential double-edged sword.^41^ Conversely, hen egg white (HEW)-derived lysozyme has previously been reported to reduce chemically induced colitis in animal models.^42^ Lysozyme is also produced by fungi, including *Acremonium alcalophilum*, with no observable bacteriolytic activity^43^ and thus considered safe to use in, e.g., animal feed.^44,45^ This fungal lysozyme belongs to the glycoside hydrolase family (GH) 25 and is thereby distinct from human and HEW lysozymes included in the GH22 family.^46^ Recently, a non-purified feed additive of *A. alcalophilum*-derived lysozyme was reported to degrade bacterial PGN^46^ to NOD2-activating muramyl dipeptides (MDP) reducing duodenal inflammation^43^ and reduce the abundance of CD45-positive cells in the ileum of broiler chickens,^47^ thus pointing toward enhanced barrier integrity.

Considering the above-described GI implications of *A. alcalophilum*-derived lysozyme, we here investigated the disease-preventing potential of a highly purified version of this lysozyme in both HFD-induced obesity-linked IR and DSS-provoked colitis. We report that purified *A. alcalophilum*-derived lysozyme protects against HFD-induced microbiota encroachment, mitigates fasting hyperinsulinemia, and further leverages the gut microbiota to alleviate DSS-induced gut inflammation. Pathway analysis of the gut microbiota revealed similar traits followed by lysozyme supplementation in both *in vivo* models, despite a vastly different microbiota community structure between the two mouse strains. These findings suggest that *A. alcalophilum* lysozyme may target shared GI manifestations in obesity and colitis.

## Results

### Lysozyme alleviates diet-induced fasting hyperinsulinemia, increases mucus production and reduces microbiota encroachment

**Figure 1.**
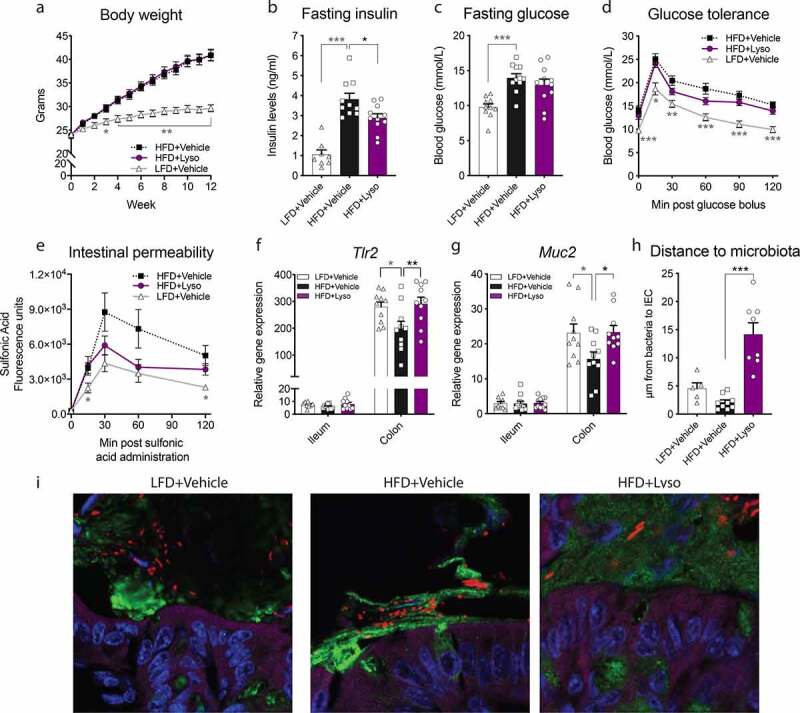
**Lysozyme administration reduces fasting insulin levels associated with normalized *Tlr2* and *Muc2* expression in HFD-fed C57BL6/J mice. a**) Weekly body weight measure in grams during the study period. **b)** 6 h fasting insulin levels in week 10 of the study period. **c)** 6 h fasting blood glucose levels in week 10 of the study. **d)** Blood glucose levels during oral glucose tolerance test (oGTT) in week 10 with 2 µg glucose per g lean mass measured by MR scans. **e)** Fluorescence measured in plasma sampled at indicated minutes post oral sulfonic acid administration. **f)** Relative gene expression of *Toll-like receptor* (*Tlr) 2* in ileum and colon tissue assessed by RT-qPCR. **g)** Relative *Mucin* (*Muc*) 2 gene expression in ileum and colon tissue assessed by RT-qPCR. **h)** Distances of the closest bacteria to intestinal epithelial cells (IEC) in proximal colon per condition over three high-powered fields per mouse, with each dot representing the average distance to bacteria per field of 2–3 representative mice per group. **i)** Representative slides of confocal microscopy analysis of microbiota localization; Muc2 (green), actin (purple), bacteria (red) and DNA (blue). Each slide measures 70 × 70 µm. **a, d, e)** Graphs depict group mean ± SEM. Repeated-measures two-way ANOVA with Geisser-Greenhouse correction and Dunnet’s multiple comparisons test to HFD+Vehicle. **b, c, f, g, h)** Graphs depict group mean ± SEM and individual data points. One-way ANOVA with Dunnet’s multiple comparisons test comparing to HFD+Vehicle. **a-h)** * = *p* < .05, ** *p* < .01, *** *p* < .001. Grey asterisk indicates significant difference between LFD+Vehicle vs HFD+Vehicle and black indicates HFD+Vehicle vs HFD+Lyso

We first investigated the potential effect of lysozyme administration in diet-induced obese mice (**Fig S1a**). Mice fed HFD for 12 weeks gained more body weight compared to LFD-fed mice, with no measurable impact from daily lysozyme supplementation on body composition or energy intake (Figure 1a**, Fig S2a-c**). Lysozyme-receiving mice did, however, exhibit lower fasting insulin levels compared to vehicle-treated counterparts (Figure 1b). Lysozyme further tended to reduce blood glucose post, but not prior, glucose challenge (two-way ANOVA RM = 0.10) compared to vehicle-treated HFD counterparts (Figure 1c-d). Still, glucose stimulated insulin concentration was unaffected by lysozyme supplementation (**Fig S2d**). Akin to diminished fasting insulin, known to modulate gut leakiness,^48^ we observed a numerical protection from HFD-induced gut permeability in lysozyme-gavaged animals (Figure 1e). Despite these *in vivo* indications of increased gut permeability in HFD-fed mice, circulating LPS, intestinal inflammation, and expression of selected tight junction genes in colon and ileum remained similar between groups (**Fig S2e-i**). Still, lysozyme normalized the HFD-induced reduction in *Tlr2* expression in colon (Figure 1f). TLR2 activation has been linked to mucus regulation by colonic goblet cells,^49^ prompting us to investigate mucus production. Lysozyme supplementation prevented HFD-induced colonic *Muc2* downregulation (Figure 1g). Moreover, such lysozyme-mediated preservation of mucus integrity was associated with a more than sixfold increase in the average distance from intestinal epithelial cells to the nearest bacteria, thus demonstrating that lysozyme supplementation sufficiently prevents microbiota encroachment induced by HFD consumption (Figure 1h-i).

### Lysozyme progressively modifies gut microbiota composition and its predicted functions

**Figure 2.**
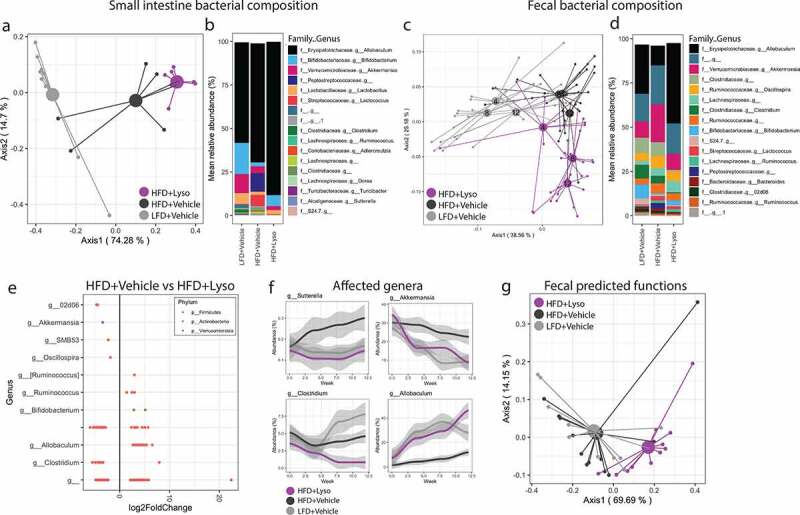
**Gut microbiota composition and predicted functionality of HFD-fed C57BL6/J mice was modified by lysozyme administration a)** Principle coordinate analysis (PCoA) of overall small intestine bacterial presence using unweighted UniFrac distances at the end of the study period sampled 3–5 h after the last lysozyme/vehicle administration. Centroids indicate group average. PERMANOVA test between LFD+Vehicle vs. HFD+Vehicle *p* = .002 and between HFD+Vehicle vs. HFD+Lyso *p* = .005. **b)** Mean relative abundance in % of 17 most abundant aggregated bacterial genera in the small intestine samples shown in a. Missing entries indicate unclassified family and/or genus. **c)** PCoA using weighted UniFrac distances of overall bacterial presence in fecal samples week 4, 8, and 12 after initiation of LFD/HFD feeding and Vehicle/Lysozyme administration, sampled approximately 24 hours since the latest lysozyme/vehicle administration. Centroids indicate group mean at the indicated week after study start. PERMANOVA test between LFD+Vehicle vs. HFD+Vehicle *p* < .01 at all sampled times. PERMANOVA test between HFD+Vehicle vs. HFD+Lyso *p* = .078 at week 4, *p* < .001 at week 8 and 12. **d)** Mean relative abundance in % of 17 most abundant aggregated bacterial genera in fecal samples in week 12 of the study. Missing entries indicate unclassified family and/or genus. **e)** ASVs with differential abundance (FDR adjusted *p* < .05) by DEseq2 analysis comparing fecal microbiota of HFD+Lysozyme to HFD+Vehicle at week 12 of the study. ASVs are categorized with their classified genus and colored by their classified phylum. Missing entries indicate unclassified genus. **f)** Relative abundance in % of genera found differentially abundant in e from baseline (0) over the study period (4–12) with similar baseline abundance between the groups and a tendency across the entire aggregated genus abundance. Graphs depict group mean and 75% CI. **g)** PCoA of predicted KEGG Orthologs in fecal samples from week 12 of the study using Bray-Curtis distance sampled approximately 24 hours since the latest lysozyme/vehicle administration. Centroids indicate group mean. PERMANOVA test between LFD+Vehicle vs HFD+Vehicle *p* = .67, HFD+Vehicle vs HFD+Lyso *p* = .006

**Figure 3.**
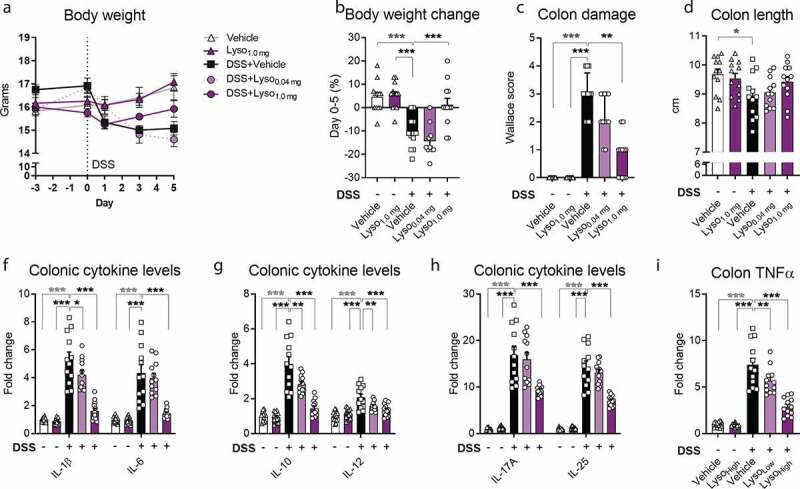
**Lysozyme dose-dependently prevents Dextran-sulfate sodium (DSS)-induced colitis in BALB/c mice a)** Body weight in grams as group mean ± SEM at baseline (day −3), at the start of DSS-challenge (day 0), and the following study period until end of the study (day 5). **b)** Body weight change during DSS-challenge from day 0 to 5 during as % of body weight. **c)** Colon damage assessed by Wallace histological scoring at the end of the study period. Bars indicate group median and interquartile range. Kruskal–Wallis test and Dunn’s multiple comparisons test to the DSS+Vehicle group. **d)** Colon length in cm. **e)** Interleukin (IL)-1β and IL-6 cytokine levels in colon tissue as fold change to vehicle group. **f)** IL-10 and IL-12 cytokine levels in colon tissue as fold change to vehicle group. **g)** IL-17A and IL-25 cytokine levels in colon tissue as fold change to vehicle group. **h)** TNFα cytokine levels in colon tissue as fold change to vehicle group. **b,d-h)** Graphs depict mean ± SEM with individual data points. One-way ANOVA with Dunnet’s multiple comparisons test to DSS+Vehicle group. **a-h)** * = *p* < .05, ** *p* < .01, *** *p* < .001. Grey asterisk indicates significant difference between Vehicle+Vehicle vs DSS+Vehicle and black indicates comparisons between DSS+Vehicle vs Lyso-supplemented groups

As diminished encroachment might reflect bactericidal activity, we next assessed longitudinal changes in the fecal microbiota of experimental mice. Microbiota composition analysis revealed similar signatures at the beginning of the study, where all mice were fed a low-fat reference diet (**Fig S3a-b**). HFD-feeding induced a vastly different microbiota composition in both small intestine and fecal samples (Figure 2a-d). Longitudinal sampling revealed instant changes to the fecal microbiota by dietary intervention, while the impact of lysozyme administration occurred gradually. Mice receiving daily doses of orally administered lysozyme thus exhibited a unique and progressive trajectory in their microbiota community structure compared to any other group (Figure 2c). Despite this progressive trajectory, several of the HFD-induced changes to the fecal, but not small intestinal, microbiota were countered by lysozyme supplementation (Figure 2a-f). The alpha diversity of the microbiota was not affected by lysozyme administration, pointing toward alterations in relative microbial abundances rather than an antimicrobial effect of this HDP (**Fig S3c-d**). In agreement with this notion, the relative fecal abundances of numerous bacterial ASVs were modified in lysozyme receiving mice compared to their vehicle-receiving counterparts. A majority of the regulated ASVs in fecal samples belonged to the Firmicutes phylum. These included a reduction of *Akkermansia muciniphila* and *Oscillospira*, while *Ruminococcus gnavus*, an unclassified species of the *Ruminococcus* genus, and *Bifidobacterium pseudolongum* and *Allobaculum* were increased in relative abundance in lysozyme receiving mice (Figure 2e). Four bacterial genera with modified ASV abundances showed a change in the entire genus followed by lysozyme supplementation (Figure 2f), while other ASV modifications appeared species dependent (**Fig S3e**). We next mapped the predicted functions of the bacterial community, revealing a significant shift in fecal but not small intestinal samples of mice receiving lysozyme (Figure 2g, Figure3f).

### Lysozyme dose-dependently prevents DSS-induced colitis

To further investigate the beneficial outcome of lysozyme supplementation on intestinal health, we assessed colon damage in chemically induced mild colitis. Mice received one of the two doses of lysozyme or vehicle from 2 days before and during the 5 days of DSS-challenge (**Fig S1b**). Mice receiving 1.0, but not 0.04, mg lysozyme were protected against DSS-induced reduction in body weight (Figure 3a-b). Despite a lack of protection against diminished bodyweight upon the DSS-challenge, 0.04 mg lysozyme effectively curbed intestinal inflammation (Figure 3c-h). We observed a highly consistent trend toward dose-dependency across all measured readouts (Figure 3a-h). A trait that was replicated in an independent study-cohort using an additional third, intermediary dose (**Fig S4a-h**). Importantly, 1.0 mg of lysozyme administered to non-DSS-challenged mice did not induce measurable changes in colonic damage or cytokine levels compared to vehicle-treated control mice, thus indicating that lysozyme alone did not affect immune homeostasis in healthy mice (Figure 3c-h, **Fig S4c-h**).

### Lysozyme partially protects against DSS-induced microbiota changes and modifies its predicted functions

**Figure 4.**
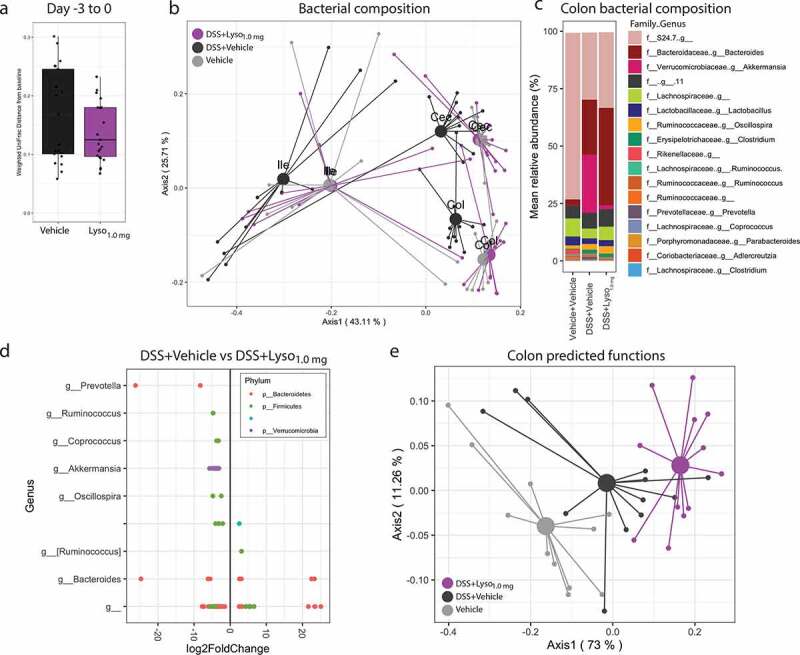
**Lysozyme partially protects against DSS-induced effects on microbiota composition in BALB/c mice a)** Weighted UniFrac distance from fecal microbiota day −3 to 0 within the same mouse. Fecal pellets were sampled prior DSS-challenge and shows the changes bacterial composition induced by administration of two days vehicle or 1 mg/day lysozyme. **b)** PCoA using weighted UniFrac distances of overall bacterial abundances in ileum (Ile), cecum (Cec), and colon (Col) samples at the end of the study in day 5 sampled a day after the latest vehicle or lysozyme administration. Centroids indicate group mean. Ileum PERMANOVA test between DSS+Vehicle vs. Vehicle+Vehicle *p* = .164, DSS+Vehicle vs. DSS+Lyso_1.0 mg_ *p* = .062. Cecum PERMANOVA test between DSS+Vehicle vs. Vehicle+Vehicle *p* = .001, DSS+Vehicle vs. DSS+Lyso_1.0 mg_ *p* = .009. Colon PERMANOVA test between DSS+Vehicle vs. Vehicle+Vehicle *p* = .001, DSS+Vehicle vs. DSS+Lyso_1.0 mg_ *p* = .001. **c)** Mean relative abundance in % of 17 most abundant aggregated bacterial genera in colon samples of the end of the study. Missing entries indicate unclassified family and/or genus. **d)** ASVs with differential abundance (FDR adjusted *p* < .05) by DEseq2 analysis comparing colon microbiota composition of DSS+Vehicle to DSS+Lyso_1.0 mg_ group at day 5 of the study sampled a day after the latest vehicle or lysozyme administration. ASVs are categorized with their classified genus and colored by their classified phylum. Missing entries indicate unclassified genus and/or phylum. **e)** PCoA of KEGG orthologs using Bray-Curtis distances based on 16S rRNA gene amplicons of colon samples week 12. Centroids indicate the mean of each group. PERMANOVA test between Vehicle+Vehicle vs. DSS+Vehicle *p* = .016 and between DSS+Vehicle vs. DSS+Lyso_1.0 mg_ *p* = .002

Healthy chow-fed mice receiving 1.0 mg of lysozyme for 2 days had similar changes in fecal microbiota composition from their baseline samples as vehicle-treated reference mice (Figure 4a). We next assessed gut microbial composition in ileum, cecum and colon at the end of the study. Consistent with previous reports, we observed pronounced inter-location variations in community structures (Figure 4b).^50^ In agreement with the colonic target of DSS-induced colitis, DSS-challenge induced consistent changes in the distal intestines but only modest changes in the ileum (Figure 4b**-c, Fig S4i-j**). Lysozyme partially protected against DSS-induced gut microbiota disturbances, further corroborated by a clear separation from vehicle receiving counterparts in the most distal intestinal locations, cecum and colon (Figure 4b-c, **Fig S4j**). Analogous to our HFD-fed obesity model, we observed a pronounced increase in numerous ASVs from the mucus degrading genus *Akkermansia* in the colon of DSS-challenged control mice (**Fig S4i**). Lysozyme protected against this DSS-related trajectory with a general decrease in several ASVs classified as *A. muciniphila* and within the *Prevotella* and *Oscillospira* genera, while we observed an increase in *R. gnavus*, hence aligning the lysozyme mediated improvements in DSS challenged mice to the traits observed in HFD-fed mice (Figure 4c-d). The predicted functions of the gut microbiota were, similar to the microbiota composition, mostly affected by lysozyme in colon samples (Figure 4e, Figure4k). We then compared the lysozyme-modified pathways based on the KEGG orthologs across the HFD- and DSS-study. To this end, we identified 21 pathways regulated in both studies of which 13 were consistently regulated by lysozyme across studies. These consistently affected pathways related to amino acid and nucleotide/nucleoside synthesis, alterations in carbohydrate metabolism, as well as three distinct pathways involving peptidoglycan synthesis and maturation (Table 1).

**Table 1.** Commonly affected predicted microbial pathways

	DIO		DSS				
BioCyc Pathway ID	log2FoldChange	padj	log2FoldChange	padj	Pathway	Expected taxonomic range	Pathway Superclass
**SER-GLYSYN-PWY**	**0.45620826**	**0.00448269**	**0.14305009**	**0.00167976**	**superpathway of L-serine and glycine biosynthesis I**	**Archaea, Bacteria, Eukaryota**	**Amino acid biosynthesis**
**PWY-5097**	**0.23420644**	**0.03977512**	**0.08283871**	**0.0009806**	**L-lysine biosynthesis VI**	**Bacteria**	**Amino acid biosynthesis**
**PWY-2942**	**0.20625525**	**0.0453016**	**0.08106705**	**0.00019338**	**L-lysine biosynthesis III**	**Bacteria**	**Amino acid biosynthesis**
**DTDPRHAMSYN-PWY**	**0.28709694**	**0.00990907**	**0.18802626**	**0.01003259**	**dTDP-beta-L-rhamnose biosynthesis**	**Archaea, Bacteria, Eukaryota**	**Carbohydrate biosynthesis**
**COLANSYN-PWY**	**−0.4548192**	**0.01514569**	**−0.4506646**	**0.01556932**	**colanic acid building block biosynthesis**	**Proteobacteria**	**Carbohydrate biosynthesis**
**PWY-6737**	**0.30662916**	**0.00990907**	**0.14879281**	**0.01304249**	**starch degradation V**	**Archaea**	**Carbohydrate degradation**
**PWY-6901**	**0.80606367**	**0.03551929**	**0.45694215**	**0.00687032**	**superpathway of glucose and xylose degradation**	**Bacteria**	**Carbohydrate degradation**
**PWY-5384**	**−0.9248353**	**0.04506064**	**−3.8620577**	**1.38E-07**	**sucrose degradation IV (sucrose phosphorylase)**	**Actinobacteria**	**Carbohydrate degradation**
**PEPTIDOGLYCANSYN-PWY**	**0.22943748**	**0.04004827**	**0.14666461**	**1.06E-05**	**peptidoglycan biosynthesis I (meso-diaminopimelate containing)**	**Bacteria**	**Cell wall biosynthesis**
**PWY-6387**	**0.23189181**	**0.04280103**	**0.17325694**	**5.35E-06**	**UDP-N-acetylmuramoyl-pentapeptide biosynthesis I (meso-diaminopimelate containing)**	**Bacteria**	**Cell wall biosynthesis**
**PWY0-1586**	**0.76138824**	**0.0453016**	**0.35571034**	**0.00227927**	**peptidoglycan maturation (meso-diaminopimelate containing)**	**Bacteria**	**Cell wall biosynthesis**
**PWY-7211**	**−0.7328499**	**0.03229654**	**−1.9612053**	**1.74E-06**	**superpathway of pyrimidine deoxyribonucleotides de novo biosynthesis**	**Archaea, Bacteria, Eukaryota**	**Deoxyribonucleotide biosynthesis**
**PWY-6123**	**0.20890499**	**0.04046209**	**0.08394989**	**0.00445139**	**inosine-5ʹ-phosphate biosynthesis I**	**Bacteria**	**Nucleoside and nucleotide biosynthesis**
**ARGSYNBSUB-PWY**	**0.2281011**	**0.02310253**	**−0.4028675**	**0.03448026**	**L-arginine biosynthesis II (acetyl cycle)**	**Bacteria**	**Amino acid biosynthesis**
**HISDEG-PWY**	**−0.7826256**	**0.0176437**	**0.57885065**	**0.04823181**	**L-histidine degradation I**	**Bacteria**	**Amino acid degradation**
**PWY-7332**	**1.08044723**	**0.00448269**	**−1.3011902**	**0.00303795**	**superpathway of UDP-N-acetylglucosamine-derived O-antigen building blocks synthesis**	**Bacteria**	**Carbohydrate biosynthesis**
**PWY-7456**	**−1.3526842**	**0.00342097**	**0.55061045**	**0.01020155**	**beta-(1,4)-mannan degradation**	**Bacteria**	**Carbohydrate degradation**
**RHAMCAT-PWY**	**−1.1220469**	**0.02519796**	**0.77143331**	**0.00280173**	**L-rhamnose degradation I**	**Bacteria**	**Carbohydrate degradation**
**PWY-7539**	**−0.3682025**	**0.00448269**	**0.23121565**	**0.0427331**	**6-hydroxymethyl-dihydropterin diphosphate biosynthesis III (Chlamydia)**	**Chlamydia**	**Folate biosynthesis**
**PWY-7199**	**−0.7381764**	**0.00366873**	**0.24402053**	**0.00218249**	**pyrimidine deoxyribonucelosides salvage**	**Bacteria**	**Nucleoside and nucleotide biosynthesis**
**PWY-5695**	**−0.687757**	**0.03488927**	**0.26807919**	**0.00035365**	**inosine-5ʹ-phosphate degradation**	**Bacteria**	**Nucleoside and nucleotide degradation**

Predicted microbial pathways significantly regulated by lysozyme (DESeq2 analysis FDR adjusted *p* < 0.05) in both HFD-fed C57BL6/J and DSS-colitis mice. Positive log2foldchange (Green) indicate increased pathways by lysozyme where negative (red) indicates decreased pathway.

### Lysozyme-mediated mitigation of DSS-induced colitis relies on a functional gut microbiota

**Figure 5.**
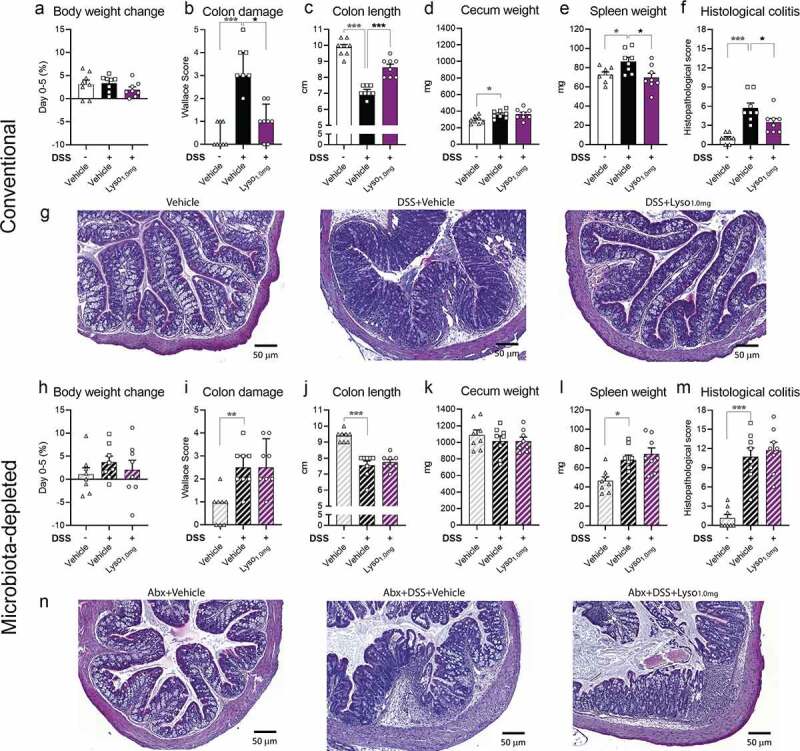
**Lysozyme leverages the gut microbiota to alleviate DSS-induced colitis in BALB/c mice a)** Body weight change during DSS-challenge from day 0 to 5 during as % of body weight in conventional mice. **b)** Colon damage in conventional mice assessed by Wallace histological scoring at the end of the study period. Bars indicate group median and interquartile range. Kruskal–Wallis test and Dunn’s multiple comparisons test to the DSS+Vehicle group. **c)** Colon length in cm in conventional mice. **d)** Cecum wet weight in mg in conventional mice. **e)** Spleen weight in grams in conventional mice. **f)** Adapted histopathological colitis scoring of H&E-stained colon slides from conventional mice shown in g. **g)** Representative H&E-stained image of colon sections from the indicated group. **h-n)** As a-g in mice receiving antibiotics (ampicillin and neomycin) in drinking water. **h)** Colon length in cm in mice receiving antibiotics. Kruskal–Wallis test and Dunn’s multiple comparisons test to the DSS+Vehicle group. **a, c-e, h, j-l)** Graphs depict mean ± SEM with individual data points. One-way ANOVA with Dunnet’s multiple comparisons test to DSS+Vehicle group. **a-f, h-m)** * = *p* < .05, ** *p* < .01, *** *p* < .001. Grey asterisk indicates significant difference between Vehicle+Vehicle vs DSS+Vehicle and black indicates comparisons between DSS+Vehicle vs Lyso-supplemented groups in both conventional mice and mice receiving antibiotics

We next sought to investigate the role played by the intestinal microbiota in the promotion of lysozyme-mediated protection against DSS-induced colitis. To this end, we subjected mice to a similar challenge and treatment regime as described above and included an additional study arm of microbiota depleted animals (**Fig S1c**), known to precipitate similar DSS-induced intestinal injury as conventional mice.^51^ Moreover, the experiment was performed in a new vivarium and with mice from a different vendor in order to ensure reproducibility of lysozyme-mediated protection against DSS-induced colitis (DSS-induced colitis is notoriously difficult to phenocopy across cohorts^52^). Albeit the DSS-challenged vehicle-treated mice in this study did not lose weight, the colitis phenotype mimicked our previous observations (Figure 5a-c). Moreover, daily lysozyme gavage conferred strong protection against DSS-induced colon shortening and spleen weight, as well as both macro- and microscopic colitis scores (Figure 5a-g). Importantly, while antibiotics themselves are not sufficient to inhibit lysozyme activity *in vitro* (**Fig S4l**), lysozyme-mediated protection was abolished in microbiota depleted mice (Figure 5h-n). These data importantly suggest that lysozyme impact on colitis is dependent on the gut microbiota.

## Discussion

In the present study, we first investigated the preventive effects of lysozyme administration on HFD-induced intestinal changes. Here, lysozyme reduced fasting insulin levels, commonly used for clinical determination of IR,^53^ despite not improving HFD-induced obesity. Diminished fasting insulin in lysozyme-gavaged mice is associated with improved barrier function and markedly diminished microbiota encroachment. These beneficial traits were accompanied by a progressive change in fecal and small intestinal microbiota composition where lysozyme administration specifically modified the predicted functions of the fecal microbiota. Encouraged by the observed benefits of lysozyme administration in the obesity setting primarily targeting the large intestine, we next evaluated the efficacy of a short-term DSS-challenged mouse model of mild colitis. Here, lysozyme is reproducibly and dose-dependently protected against DSS-induced colon damage and mitigated disease-associated microbiota changes in cecum and colon. The lysozyme-induced alterations in the predicted functions of the gut microbiota shared numerous traits between the models, despite differences in sex, mouse strain, vendor and experimental facility. We further reproduced the DSS-induced colitis model in a new vivarium, where groups of antibiotic-treated mice were also included. Depletion of the commensal microbiota by broad-spectrum antibiotics abrogated lysozyme efficacy, pointing toward microbiota-dependency in the lysozyme mediated protection against DSS-induced colitis. Collectively, these observations corroborate consistent and biologically relevant adaptations in the fecal microbiota of lysozyme-gavaged mice.

**Figure 6.**
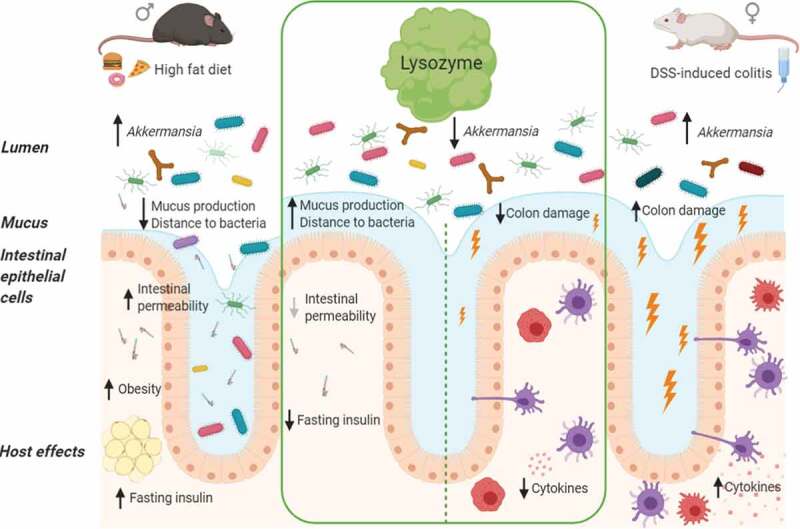
**Lysozyme effects in HFD-fed C57BL/6 J and DSS-induced colitis BALB/c mice** HFD and DSS induced intestinal changes in both used mouse models. Despite changes in study design, phenotypes, sex, mouse strain, diet, lysozyme dosage and duration, and experimental facility lysozyme ameliorated both diet- and DSS-induced phenotypes. This included consistent changes in several bacterial abundances and the predicted microbial functions

Although rarely studied in parallel, HFD-induced intestinal deterioration and DSS-induced colitis share common features in etiology, including changes in gut microbiota composition and microbiota encroachment^15,54^ (Figure 6). In both disease models, the relative *A. muciniphila* abundances were negatively associated with gut health markers. *A. muciniphila* is a mucus-degrading species and generally considered a beneficial microbe able to protect against both HFD-induced hyperinsulinemia^55^ and DSS-induced colitis.^56^ Despite the widely accepted benefits of *A. muciniphila*, numerous studies also challenge this binary view reporting significant colitis-associated blooms in *Akkermansia* abundances.^52,57–60^ The species has further been shown to exaggerate proton pump inhibitor (PPI)-induced small intestinal injury by diminishing the jejunal mucus layer, hence enhancing inflammatory burden^61^ and promoting IL-1a secretion from colonic tissue.^62,63^ Such reports are in agreement with observations from graft versus host diseases, similarly characterized by substantial gut disturbances.^64^ In our study, we observed a robust induction of *A. muciniphila* in vehicle treated HFD-fed and DSS-challenged control mice, an increase that was fully prevented by lysozyme in both models. Corroborating a causal link between lysozyme administration and *A. muciniphila* regulation, *Lyz1* knockout mice, incapable of mounting an endogenous lysozyme response, were recently reported to exhibit increased *Akkermansia* abundances.^40^ Although bacterial (i.e. *Akkermansia*) induced mucus degradation might stimulate renewal, hence improving barrier function, in mild low-grade inflammatory diseases,^65^ the same traits might facilitate detrimental outcomes during more severe gut inflammation as observed in experimental colitis and immunocompromised individuals.^66^ Still, it remains elusive if lysozyme administration inhibits further growth or actively degrades mucolytic bacteria, such as *Akkermansia*. Future studies are therefore warranted to elucidate if such degradation takes place and to what extent it would liberate bioactive compounds from *Akkermansia* to mitigate gut inflammation.^67^

Intriguingly, a recent study elegantly demonstrated how endogenous lysozyme, when abundantly expressed in the colon of IBD patients, degraded colonic *R. gnavus*, thereby liberating its proinflammatory polysaccharide^68^ exaggerating intestinal inflammation.^40^ This novel finding presents endogenous lysozyme as a double-edged sword^41^ that may be tweaked by inflammatory status and microbiota composition. To this end, lysozyme-mediated lysis of *Lactococcus lactis* facilitates delivery of colitis-attenuating superoxide dismutase to the inflamed colon.^69^ Such descriptions add complexity to the otherwise well-described HDP-mediated improvements in barrier function to protect against bacterial translocation.^11^ Peculiarly, we observed increased *R. gnavus* abundances in both disease models following lysozyme administration. Our data on dose-dependent downregulation of intestinal inflammation are thus in sharp contrast to the recent report by Yu *et al*.^40^ Future studies are thus warranted to elucidate if the mentioned discrepancy relates to differences in sources (endogenous versus *A. alcalophilum*-derived) and/or family (GH22 vs GH25 lysozymes), microbial community structure or inflammatory tone upon lysozyme administration.

In conclusion, *A. alcalophilum-derived* lysozyme administration reshaped the gut microbiota composition along with its predicted functions, diminished HFD-induced intestinal permeability and bacterial encroachment, as well as microbiota-dependently diminishing DSS-induced colitis. The beneficial impact across two different mouse strains, in both sexes, combined with the immune regulating properties reported in broiler chickens,^47,70^ merits further investigation to assess its prospects of clinical translatability.

## Materials and methods:

### Animals

Male C57BL6/J mice (Jackson) or female BALB/c mice (Charles River) of 6 weeks of age were acclimatized for 2 weeks on either compositional-defined low-fat diet (D12450H, Research Diets) or standard, fiber-rich, chow diet (RM1P diet, Special Diet Services (First DSS studies), or LabDiet 5021 (DSS-study including antibiotics)), respectively. All mice were co-housed with free access to water and 12 hours light–dark cycle at room temperature.

C57BL6/J mice were then switched to a compositional defined HFD (D12451, Research Diets) or kept on the matched LFD for the duration of 12 weeks while receiving daily oral gavage 4 h ± 30 min into the light cycle of 100 µL PBS (Gibco) with or without 0.5 mg lysozyme from *A. alcalophilum* grown in the production organism *Trichoderma reesei*, as previously described^44^ and further purified^46^ to a purity of >95%; endotoxins <0.004 EU/mL. Body weights were monitored weekly and feed intake measured and exchanged thrice a week during the study period, while body composition by magnetic resonance (MR) scans (Minispec LF90, Bruker) and fecal sampling was assessed every 4 weeks. After 12 weeks, the mice were anesthetized with isoflurane (Fresenius Kabi) after 6 h fasting from 1 h into the light cycle with their usual daily gavage 4 h into the light cycle and euthanized by cardiac puncture followed by cervical dislocation. See study outline **Fig S1a**. The study in C57BL6/J mice was conducted in accordance with the Canadian Council on Animal Care guidelines and regulations approved by Laval University, Canada (license 2017–086-1).

After acclimatization, BALB/c mice received a daily oral gavage 3.5 h into the light cycle of 200 µL PBS (Gibco) with or without 0.04 mg or 1 mg *A. alcalophilum-*derived lysozyme for 7 days to the day before euthanasia while continuously fed chow diet. After 2 days of daily lysozyme or vehicle administration colitis was induced in selected groups by addition of DSS in the drinking water (3%w/v, Product code 42867, Sigma Aldrich) for the following 5 days until euthanasia. Body weights were monitored daily. At day 5 of DSS-challenge mice were anesthetized with isoflurane (UK National Veterinary Service Ltd) and euthanized by cardiac puncture followed by pentobarbitone (UK National Veterinary Service Ltd) overdose and cervical dislocation. See study outline **Fig S1b**. The primary outcomes of the BALB/c study were reproduced in an identical study setup adding an intermediary dose of 0.2 mg lysozyme. The studies in BALB/c mice were conducted with relevant guidelines and regulations approved by the UK Home Office Scientific Procedures Act under project license 80/2613. The BALB/c studies were further reproduced in a different study facility including the 1.0 mg lysozyme dose where half of the mice were administered antibiotics in the form of ampicillin (1.0 g/L) and neomycin (0.5 g/L) in the drinking water. Antibiotics were administered from 5 days prior to the first administration of lysozyme and/or 7 days prior to the addition of DSS, as outlined in **Fig S1c**, and were otherwise identical to the study outlined in Figure1b. The antibiotic study followed the relevant guidelines and regulations approved by the French Ministry of Research and Innovation under license APAFIS#24788-2019102806256593 v8.

### Oral glucose tolerance test (oGTT), glucose-stimulated insulin concentration (GSIC), and intestinal permeability assay

An oGTT and an sulfonic acid assay for intestinal permeability were carried out in week 10 of the experimental protocol in C57BL6/J mice. Mice were fasted 6 h from 2 h into the light cycle and gavaged with their usual daily supplementation at 4 h into the light cycle. Fasting blood glucose was measured and plasma insulin sampled from the tail vein prior to oral gavage with 2 µg/g lean mass dextrose (Hospira) and 1.5 mg fluorescein-5 (6)-sulfonic acid (Invitrogen) dissolved in 150 μL suspension of 0.5% Carboxymethylcellulose Sodium Salt (CMC) (Sigma) in distilled water. Blood glucose was measured at 0, 15, 30, 60, 90, and 120 min after dextrose challenge, and blood samples for quantification of plasma insulin, and sulfonic acid levels were sampled in EDTA-prepared capillary tubes (Sarstedt) at 0, 15, 30, 60, and 120 min postgavage. Mice were subcutaneously injected with 0.5 mL saline (Hospira) after the procedure for rehydration. Blood samples were centrifuged for 10 min at 1000 rcf at 4°C. Plasma insulin was quantified by Mouse Ultrasensitive Insulin ELISA (Alpco) following the manufacturer’s protocol. Gut permeability was assessed by quantification of fluorescence from the sulfonic acid fluorescein in 5 µL plasma transferred to black 96-well optical-bottom plates (Nunc) and kept on wet ice protected from light until the addition of 150 μL of 0.5% CMC in distilled water and read at excitation/emission 485/528 nm wavelength.

### Tissue cytokine quantification

Snap-frozen liver, ileum, and colon tissue from C57BL6/J mice were cryo-grinded in liquid nitrogen and washed by adding 200 µL cooled PBS added protease inhibitors (Sigma-Aldrich), centrifuged at 16,000 rcf for 20 sec at 4°C and the liquid aspirated. For liver tissue, the wash-step was repeated 10 times, while intestinal tissues were washed 2 times. Afterward, 400 µL T-PER Tissue Protein Extraction Buffer (ThermoFisher) and beads were added and vortexed on TissueLyzer (Qiagen) for 2 × 1 min at 50 os/sec. Samples were centrifuged at 4,000 rcf for 1 min at 4°C and the supernatant transferred to new tubes that were then centrifuged again 13,000 rcf for 10 sec at 4°C. Protein extracted from intestinal tissue was filtered through 0.22 µm centrifugal filters (Merck-Millipore) and centrifuged 12,000 rcf for 4 min. Protein concentration was measured in triplicates using Pierce BCA Protein Assay kit (ThermoFisher) using the manufacturer’s instructions. Cytokine levels in 50 µg extracted protein were measured using multiplex assays (Merck Millipore for liver and colon, BioRad for ileum) using a Bio-plex Multiplex System (BioRad). Colon tissue from BALB/c mice was placed in a lysing tube containing lysis solution added protease inhibitor and tissue protein extraction reagent at a ratio of 1 g of tissue to 5 mL lysis solution. The tissue was homogenized 3 times at 4247 rcf for 30 sec after which the samples were centrifuged at 92 rcf for 5 min at 4°C to extract the protein. The supernatants were evaluated using a multiplex assay (Merck Millipore) for a range of Th1 and Th17 characteristic cytokines using a Magpix system (Luminex).

### Lipopolysaccharide (LPS)

Blood from euthanasia of C57BL6/J mice was sampled with EDTA-coated syringes and immediately kept on ice and centrifuged for 10 min at 1000 rcf at 4°C and the plasma stored at −80°C until further processing. LPS was quantified by ELISA (MyBioSource) following the manufacturer’s instructions.

### Quantitative reverse transcriptase PCR (qPCR) of intestinal tissues

RNA from snap-frozen ileum and colon tissues from C57BL/6 J mice were extracted using the Directzol RNA Miniprep kit (Zymo Research). cDNA synthesis was made from 2 µg ileum or 1.5 µg colon RNA using High Capacity cDNA Reverse Transcriptase kit (Applied Bioscience) following the manufacturer’s protocol. qPCR was carried out using 4 µl of cDNA, 5 µl of Advanced qPCR MasterMix (Wisent Bioproducts) and 0.5 µl of each primer (diluted at a concentration of 10 µM) in a total reaction volume of 10 µL with the following cycle setting: 95°C for 2 minutes (95°C for 20 sec, 61.5–62°C for 20 sec, 72°C for 20 sec) x 40 ending with a melting Curve: 65°C to 95°C. Each target in each tissue was evaluated and accepted in the case of a unified peak from melting curves and an efficiency of 100%±15 and R^2^ > 0.95 from a standard curve. Relative expression was calculated by 2^ΔCq^ of target Cq to 18S Cq of the sample accepting replicates with coefficient of variation <0.05. Target primer sequences and annealing temperatures are found in Supplementary Table 1.

### *Immuno-fluorescent* in situ *hybridization (FISH) of mucins and localization of bacteria*

Mucus immunostaining was paired with FISH to analyze the localization of bacteria at the surface of the intestinal mucosa, as previously described.^71^ Briefly, proximal colon tissue was places in Carnoy’s solution (60% methanol, 30% chloroform, 10% glacial acid) and washed in methanol 2 × 30 min, ethanol 2 × 15 min, ethanol/xylene (1:1) 15 min, and xylene 2 × 15 min, followed by embedding in paraffin. Sections of 5 µm were made and dewaxed by preheating for 10 min at 60°C, xylene for 10 min at 60°C, and 99.5% ethanol for 10 min. Hybridization was carried out overnight at 50°C with EUB338 probe (5ʹ-GCTGCCTCCCGTAGGAGT-3ʹ, with a 5ʹ labeling using Alexa 647) diluted to a final concentration of 10 μg/mL in hybridization buffer (20 mM Tris–HCl, pH 7.4, 0.9 M NaCl, 0.1% SDS, 20% formamide). After 10 min in wash buffer (20 mM Tris–HCl, pH 7.4, 0.9 M NaCl) and 3 × 10 min in PBS, we used PAP pen (Sigma-Aldrich) to mark the section and added block solution of 5% fetal bovine serum in PBS for 30 min at 4°C. The primary antibody for Mucin-2 (MUC2) (rabbit H-300, Santa Cruz Biotechnology) was diluted 1:1500 in block solution and added overnight at 4°C. Washing was done by 3 × 10 min in PBS, after which block solution containing anti-rabbit Alexa 488 secondary antibody diluted 1:1500, Phalloidin-Tetramethylrhodamine B isothiocyanate (Sigma) at 1 μg/mL and Hoechst 33258 (Sigma) at 10 μg/mL was applied to the section for 2 h. Washing 3 × 10 min in PBS was then followed by mounting using Prolong anti-fade mounting media (Life Technologies). Observations were performed with a Zeiss LSM 700 confocal microscope with software Zen 2011 version 7.1. This software was used to determine the distance between bacteria of the microbiota and epithelial cell monolayer.^72^

### Microbiota profiling using 16S rRNA gene amplicon sequencing

Fecal samples from C57BL6/J mice were collected before the study started and at 4, 8, and 12 weeks of the study and small intestine content from the necropsy. Fecal samples from BALB/c mice were collected prior the study start (day −3) and 2 days after vehicle or lysozyme administration started prior to the DSS-challenge (day 0). The contents of the ileum, cecum, and colon were collected at necropsy (day 5). DNA was extracted using the Nucleospin Soil Extraction Kit (Machery-Nagel) on heat-inactivated samples by 15 min of heating to 95°C. 16S rRNA gene amplification targeting the V3-V4 region using forward primer S-D-Bact-0341-b-S-17 and reverse primer S-D-Bact-0785-a-A-21 with Illumina adapters^73^ (C57BL6/J study) and forward primer 341–2FDI and reverse primer 805.2RDI (BALB/c study). 16S primer sequences can be found in **Supplementary Table 2**. 16S rRNA gene amplicons were indexed using Nextera XT index kit v2 (Illumina). Libraries were cleaned using Agencourt AMPure XP beads (Beckman Coulter) and sequenced using an Illumina MiSeq desktop sequencer using the MiSeq Reagent Kit V2 (Illumina) for 2 × 250 (C57BL6/J study) or V3 for 2 × 300 (BALB/c study) bp paired-end. The generation of an amplicon sequence variant (ASV) table was done with usearch version 10.0.240.^74^ Primer binding regions were removed with fastx_truncate and reads were filtered to contain less than one error per read. The quality filtered reads were denoised with unoise3. ASV abundance was calculated by mapping with usearch global using a 97% identity threshold. The phylogenetic tree was made by aligning the 16S sequences with mafft, and the tree was inferred by FastTree. Taxonomical classification was done with the qiime classifier (qiime2-2019.4) trained on the Silva database (Silva_132). Analysis of microbiota composition was carried out in R version 4.0.2 and R Studio version 1.3.1056 using the phyloseq, vegan, and DESeq2 packages^75–78^ on nonrarefied data, including samples with a minimum of 8,000 reads. The dataset had a median of 19,035 and a mean of 20,968 reads per sample with a standard deviation of 8,414. Alpha diversity was calculated based on rarefied data to 8,000 reads per sample (**Fig S1c-d**). Predicted functionality of the gut microbiota was assessed using PICRUSt2 generated Kyoto Encyclopedia of Genes and Genomes (KEGG) orthologs and pathways.^79^

### Histological scoring of colon

Inflammation grading of the colon of BALB/c mice was performed macroscopically using a light microscope and was conducted by two blinded observers based on the Wallace scoring method. Criteria for the scoring of colonic damage were: 0) no damage; 1) Hyperemia. Normal bowel wall. No ulcers; 2) Hyperemia and thickening of bowel wall. No ulcers; 3) One ulcer without thickening of the bowel wall; 4) Two or more sites of ulceration or inflammation; 5) Two or more major sites of ulceration and inflammation or one site of ulceration/inflammation extending >1 cm along the length of the colon; 6–10) Covering damage >2 cm along the length of the colon. The score was increased by 1 for each additional centimeter of colon damage.

### H&E Staining of colonic tissue and histopathologic analysis

Following euthanasia, 1 cm of the proximal mouse colons were fixed in 10% buffered formalin for 24 hours at room temperature and then embedded in paraffin. Tissues were sectioned at 5-mm thickness and stained with hematoxylin and eosin (H&E) using standard protocols. H&E stained slides were next scored. Each colon was assigned four scores based on the degree of epithelial damage and inflammatory infiltrate in the mucosa, submucosa and muscularis/serosa, as previously described.^80^ A slight modification was made to this scoring system as follows: each of the four scores was multiplied by 1 if the change was focal, 2 if it was patchy and 3 if it was diffuse. The 4 individual scores per colon were added, resulting in a total scoring range of 0–36 per mouse.^81^

### Lysozyme activity assay in the presence and absence of antimicrobials

*A. alcalophilum*-derived lysozyme was added to an assay plate containing fluorescent-labeled PGN from *Micrococcus lysodeikticus* (EnzChek Lysozyme Assay Kit, Invitrogen) with or without antibiotics (10 mg/mL neomycin and 20 mg/mL ampicillin) and incubated at 37 degrees for 2 hours. The fluorescence signal (excitation/emission of 485/520 nm) was continuously measured and is proportional to the enzymatic activity. Assays were conducted with lysozyme concentration ranging from 0.0001 to 2.5 mg/mL. The data is represented as the normalized linear curve where each data point represents the mean from three independent measurements.

## Statistical analysis

GraphPad Prism 9 was used to calculate the statistical analysis except for the analysis of the microbiota composition described in the designated section. Each dataset was tested for normality by D’Agostino & Pearson test. Datasets not passing normality (*p* < .05) were log transformed prior testing where specific tests are described in figure legends. All repeated-measure ANOVAs include the Geisser-Greenhouse correction for adjusting for lack of sphericity. Statistical significance was considered at *p* < .05. In the study of C57BL6/J mice statistically significant differences between HFD+Vehicle and LFD+Vehicle are indicated by gray asterisks and difference between HFD+Vehicle and HFD+Lyso by black asterisks. This study includes n = 10 mice in LFD+Vehicle group, n = 11 in HFD+Vehicle and HFD+Lyso groups. The two first studies of BALB/c mice include n = 12 mice per group. The study featuring antibiotic-treated mice includes n = 8 mice per group.

## Supplementary Material

Supplemental Material

Supplemental Material

## Data Availability

All data and R scripts generated and analyzed in this manuscript are available from the corresponding author upon reasonable request.

## References

[1] Volynets V, Louis S, Pretz D, Lang L, Ostaff MJ, Wehkamp J, Bischoff SC. Intestinal barrier function and the gut microbiome are differentially affected in mice fed a western-style diet or drinking water supplemented with fructose. J. Nutr. 2017;147:770–20. doi:10.3945/jn.116.242859. 28356436

[2] Lam YY, Ha CWY, Hoffmann JMA, Oscarsson J, Dinudom A, Mather TJ, Cook DI, Hunt NH, Caterson ID, Holmes AJ, *et al*. Effects of dietary fat profile on gut permeability and microbiota and their relationships with metabolic changes in mice. Obesity. 2015;23(7):1429–1439. doi:10.1002/oby.21122. 26053244

[3] Gruber L, Kisling S, Lichti P, Martin F-P, May S, Klingenspor M, Lichtenegger M, Rychlik M, Haller D. High fat diet accelerates pathogenesis of murine crohn’s disease-like ileitis independently of obesity. PLoS One. 2013;8(8):1–13. doi:10.1371/journal.pone.0071661. PMC3745443 23977107

[4] Jensen BAH, Nielsen TS, Fritzen AM, Holm JB, Fjære E, Serup AK, Borkowski K, Risis S, Pærregaard SI, Søgaard I, *et al*. Dietary fat drives whole-body insulin resistance and promotes intestinal inflammation independent of body weight gain. Metabolism. 2016;65(12):1706–1719. doi:10.1016/j.metabol.2016.09.002. 27832859

[5] Ridaura, Vanessa K.Faith, Jeremiah J.Rey, Federico E.Knight, RobGordon, Jeffrey I. The effect of diet on the human gut microbiome: a metagenomic analysis in humanized gnotobiotic mice. Sci. Transl. Med. 2009;1:6ra14–6ra14. 10.1126/scitranslmed.3000322 PMC2894525 20368178

[6] Bisanz JE, Upadhyay V, Turnbaugh JA, Ly K, Turnbaugh PJ. Diet induces reproducible alterations in the mouse and human gut microbiome. bioRxiv. 2019:541797. doi:10.1101/541797. PMC6708278 31324413

[7] Pedersen HK, Gudmundsdottir V, Nielsen HB, Hyotylainen T, Nielsen T, Jensen BAH, Forslund K, Hildebrand F, Prifti E, Falony G, *et al*. Human gut microbes impact host serum metabolome and insulin sensitivity. Nature. 2016;535(7612):376–381. doi:10.1038/nature18646. 27409811

[8] Depommier C, Everard A, Druart C, Plovier H, Van Hul M, Vieira-Silva S, Falony G, Raes J, Maiter D, Delzenne NM, *et al*. Supplementation with Akkermansia muciniphila in overweight and obese human volunteers: a proof-of-concept exploratory study. Nat. Med. 2019;25(7):1096–1103. doi:10.1038/s41591-019-0495-2. 31263284 PMC6699990

[9] Gurung M, Li Z, You H, Rodrigues R, Jump DB, Morgun A, Shulzhenko N. Role of gut microbiota in type 2 diabetes pathophysiology. EBioMedicine. 2020;51(102590):102590. doi:10.1016/j.ebiom.2019.11.051. 31901868 PMC6948163

[10] Cani PD, Amar J, Iglesias MA, Poggi M, Knauf C, Bastelica D, Neyrinck AM, Fava F, Tuohy KM, Chabo C, *et al*. Metabolic endotoxemia initiates obesity and insulin resistance. Diabetes. 2007;56(7):1761–1772. doi:10.2337/db06-1491. 17456850

[11] Bevins CL, Salzman NH. Paneth cells, antimicrobial peptides and maintenance of intestinal homeostasis. Nat. Rev. Microbiol. 2011;9(5):356–368. doi:10.1038/nrmicro2546. 21423246

[12] Zhong H, Ren H, Lu Y, Fang C, Hou G, Yang Z, Chen B, Yang F, Zhao Y, Shi Z, *et al*. Distinct gut metagenomics and metaproteomics signatures in prediabetics and treatment-naïve type 2 diabetics. EBioMedicine. 2019;47:373–383. doi:10.1016/j.ebiom.2019.08.048. 31492563 PMC6796533

[13] Hodin, Caroline M., Verdam, Froukje J, Grootjans, Joep, Rensen, Sander S, Verheyen, Fons K, Dejong, Cornelis H C, Buurman, Wim A, Greve, Jan Willem, Lenaerts, K. Reduced Paneth cell antimicrobial protein levels correlate with activation of the unfolded protein response in the gut of obese individuals. 2011;225(2):276–284. 10.1002/path.2917 21630271

[14] Jensen BA, Marette A. Microbial translocation in type 2 diabetes: when bacterial invaders overcome host defence in human obesity. Gut. 2020;69(10):1724–1726. doi:10.1136/gutjnl-2020-321288. 32518079

[15] Chassaing B, Raja SM, Lewis JD, Srinivasan S, Gewirtz AT. Colonic microbiota encroachment correlates with dysglycemia in humans. Cell. Mol. Gastroenterol. Hepatol. 2017;4(2):205–221. doi:10.1016/j.jcmgh.2017.04.001. 28649593 PMC5472192

[16] Anhê FF, Jensen BAH, Varin TV, Servant F, Van Blerk S, Richard D, Marceau S, Surette M, Biertho L, Lelouvier B, *et al*. Type 2 diabetes influences bacterial tissue compartmentalisation in human obesity. Nat. Metab. 2020;2(3):233–242. doi:10.1038/s42255-020-0178-9. 32694777

[17] Massier L, Chakaroun R, Tabei S, Crane A, Didt KD, Fallmann J, von Bergen M, Haange S-B, Heyne H, Stumvoll M, *et al*. Adipose tissue derived bacteria are associated with inflammation in obesity and type 2 diabetes. Gut. 2020;69(10):1796–1806. doi:10.1136/gutjnl-2019-320118. 32317332

[18] Wehkamp J, Salzman NH, Porter E, Nuding S, Weichenthal M, Petras RE, Shen B, Schaeffeler E, Schwab M, Linzmeier R, *et al*. Reduced Paneth cell alpha-defensins in ileal Crohn’s disease. Proc. Natl. Acad. Sci. U. S. A. 2005;102(50):18129–18134. doi:10.1073/pnas.0505256102. 16330776 PMC1306791

[19] Wehkamp J, Koslowski M, Wang G, Stange EF. Barrier dysfunction due to distinct defensin deficiencies in small intestinal and colonic Crohn’s disease. Mucosal Immunol. 2008;1(Suppl S1):S67–S74. doi:10.1038/mi.2008.48. 19079235

[20] Ambrose NS, Johnson M, Burdon DW, Keighley MRB. Incidence of pathogenic bacteria from mesenteric lymph nodes and ileal serosa during Crohn’s disease surgery. Br. J. Surg. 1984;71(8):623–625. doi:10.1002/bjs.1800710821. 6743986

[21] Peyrin-Biroulet L, Gonzalez F, Dubuquoy L, Rousseaux C, Dubuquoy C, Decourcelle C, Saudemont A, Tachon M, Béclin E, Odou M-F, *et al*. Mesenteric fat as a source of C reactive protein and as a target for bacterial translocation in Crohn’s disease. Gut. 2012;61(1):78–85. doi:10.1136/gutjnl-2011-300370. 21940721 PMC3230831

[22] Gadaleta RM, van Erpecum KJ, Oldenburg B, Willemsen ECL, Renooij W, Murzilli S, Klomp LWJ, Siersema PD, Schipper MEI, Danese S, *et al*. Farnesoid X receptor activation inhibits inflammation and preserves the intestinal barrier in inflammatory bowel disease. Gut. 2011;60(4):463–472. doi:10.1136/gut.2010.212159. 21242261

[23] Ni J, Wu GD, Albenberg L, Tomov VT. Gut microbiota and IBD: causation or correlation? Nat. Rev. Gastroenterol. Hepatol. 2017;14(10):573–584. doi:10.1038/nrgastro.2017.88. 28743984 PMC5880536

[24] S J Ott, M Musfeldt, D F Wenderoth, J Hampe, O Brant, U R Fölsch, K N Timmis, S Schreiber. Reduction in diversity of the colonic mucosa associated bacterial microflora in patients with active inflammatory bowel disease. Gut. 2004;53(5):685–693. doi:10.1136/gut.2003.025403. 15082587 PMC1774050

[25] Hyun C-K. Molecular and pathophysiological links between metabolic disorders and inflammatory bowel diseases. Int. J. Mol. Sci. 2021;22(8). doi:10.3390/ijms22179139. PMC8430512 34502047

[26] Walters WA, Xu Z, Knight R. Meta-analyses of human gut microbes associated with obesity and IBD. FEBS Lett. 2014;588(22):4223–4233. doi:10.1016/j.febslet.2014.09.039. 25307765 PMC5050012

[27] Greenblum S, Turnbaugh PJ, Borenstein E. Metagenomic systems biology of the human gut microbiome reveals topological shifts associated with obesity and inflammatory bowel disease. Proc. Natl. Acad. Sci. 2012;109(2):594–599. doi:10.1073/pnas.1116053109. 22184244 PMC3258644

[28] Chen L, *et al*. Gut microbial co-abundance networks show specificity in inflammatory bowel disease and obesity. Nat. Commun 2020;11(4018). doi: 10.1038/s41467-020-17840-y. PMC7419557 32782301

[29] Ogura Y, Bonen DK, Inohara N, Nicolae DL, Chen FF, Ramos R, Britton H, Moran T, Karaliuskas R, Duerr RH, *et al*. Ogura 2001. Nature. 2001;411(6837):603–606. doi:10.1038/35079114. 11385577

[30] Denou, E., Lolmede, K., Garidou, L., Pomie, C., Chabo, C., Lau, Trevor C., Fullerton, Morgan D, Nigro, G., Zakaroff-Girard, A., *et al.* Defective NOD2 peptidoglycan sensing promotes diet-induced inflammation, dysbiosis, and insulin resistance. EMBO Mol. Med. 2015;7(3):259–274. doi:10.15252/emmm.201404169. 25666722 PMC4364944

[31] Bregenzer N, Hartmann A, Strauch U, Schölmerich J, Andus T, Bollheimer CL. Increased insulin resistance and beta cell activity in patients with Crohn’s disease. Inflamm. Bowel Dis. 2006;12(1):53–56. doi:10.1097/01.MIB.0000195975.97673.f5. 16374259

[32] Principi M, Iannone A, Losurdo G, Mangia M, Shahini E, Albano F, Rizzi SF, La Fortezza RF, Lovero R, Contaldo A, *et al*. Nonalcoholic fatty liver disease in inflammatory bowel disease: prevalence and risk factors. Inflamm. Bowel Dis. 2018;24(7):1–8. doi:10.1093/ibd/izy051. 29688336

[33] Harper JW, Zisman TL. Interaction of obesity and inflammatory bowel disease. World J. Gastroenterol. 2016;22(35):7868. doi:10.3748/wjg.v22.i35.7868. 27672284 PMC5028803

[34] Relationship(s SA. between obesity and inflammatory bowel diseases: possible intertwined pathogenic mechanisms. Clin. J. Gastroenterol. 2020;13(2):139–152. doi:10.1007/s12328-019-01037-y. 31452062 PMC7101293

[35] Larsen, Ida Søgaard., Fritzen, Andreas Mæchel., Carl, Christian Strini., Agerholm, Marianne., Damgaard, Mads Thue Fejerskov., Holm, Jacob Bak, Marette, André., Nordkild, Peter, Kiens., Bente, Kristiansen., Karsten Wehkamp, Jan., Jensen, Benjamin Anderschou Holbech. Human Paneth cell α-defensin-5 treatment reverses dyslipidemia and improves glucoregulatory capacity in diet-induced obese mice. Am. J. Physiol. Metab 2019;317:E42–E52. 10.1152/ajpendo.00019.2019 30860877

[36] Koeninger L, Armbruster NS, Brinch KS, Kjaerulf S, Andersen B, Langnau C, Autenrieth SE, Schneidawind D, Stange EF, Malek NP, *et al*. Human β-defensin 2 mediated immune modulation as treatment for experimental colitis. Front. Immunol. 2020;11(93). doi:10.3389/fimmu.2020.00093. PMC7006816 32076420

[37] Fleming A. On a Remarkable Bacteriolytic Element found. Proc. R. Soc. B. 1922;93:306–317.

[38] Tanaka M, Saito H, Kusumi T, Fukuda S, Shimoyama T, Sasaki Y, Suto K, Munakata A, Kudo H. Spatial distribution and histogenesis of colorectal paneth cell metaplasia in idiopathic inflammatory bowel disease. J. Gastroenterol. Hepatol. 2001;16(12):1353–1359. doi:10.1046/j.1440-1746.2001.02629.x. 11851832

[39] Meyer K, Gellhorn A, Prudden JF, Lehman WL, Steinberg A. Lysozyme activity in ulcerative alimentary disease. Am. J. Med. 1948;5(4):496–502. doi:10.1016/0002-9343(48)90100-4. 18886575

[40] Yu S, Balasubramanian I, Laubitz D, Tong K, Bandyopadhyay S, Lin X, Flores J, Singh R, Liu Y, Macazana C, *et al*. Paneth cell-derived lysozyme defines the composition of mucolytic microbiota and the inflammatory tone of the intestine. Immunity. 2020;53(2):398–416.e8. doi:10.1016/j.immuni.2020.07.010. 32814028 PMC7461615

[41] Zigdon M, Lysozyme: BS, Double-Edged A. Sword in the Intestine. Trends Immunol. 2020;xx:3–5. 10.1016/j.it.2020.10.010 33158739

[42] Lee, Maggie., Kovacs-Nolan, Jennifer., Yang, Chengbo., Archbold, Tania., Fan, Ming Z., Mine, Y. Hen egg lysozyme attenuates inflammation and modulates local gene expression in a porcine model of Dextran Sodium Sulfate (DSS)-induced colitis. J. Agric. Food Chem. 2009;57(6):2233–2240. doi:10.1021/jf803133b. 19231858

[43] Wang, Ya., Goossens, Evy., Eeckhaut, Venessa., Pérez Calvo, Estefania., Lopez-Ulibarri, Rual., Eising, Irene., Klausen, Mikkel., Debunne, Nathan., De Spiegeleer, Bart., Ducatelle, Richard., Van Immerseel, Filip. Dietary muramidase degrades bacterial peptidoglycan to NOD-activating muramyl dipeptides and reduces duodenal inflammation in broiler chickens. Br. J. Nutr 2020;53:1–27. 10.1017/S0007114520004493 33172510

[44] Lichtenberg J, Perez Calvo E, Madsen K, Østergaard Lund T, Kramer Birkved F, van Cauwenberghe S, Mourier M, Wulf-Andersen L, Jansman AJM, Lopez-Ulibarri R, *et al*. Safety evaluation of a novel muramidase for feed application. Regul. Toxicol. Pharmacol. 2017;89:57–69. doi:10.1016/j.yrtph.2017.07.014. 28720348

[45] Schliffka W, Zhai H-X, Pérez Calvo E, van Cauwenberghe S, Walsh MC, Lopez-Ulibarri R. Safety and efficacy evaluation of a novel dietary muramidase for swine. Heliyon. 2019;5(10):1–9. doi:10.1016/j.heliyon.2019.e02600. PMC6820093 31687489

[46] Frederiksen, Carsten Østergaard., Cohn, Marianne Thorup., Skov, Lars Kobberøe., Schmidt, Esben Gjerløff Wedebye., Schnorr, Kirk Matthew., Buskov, Steen., Leppänen, Miika., Maasilta, Ilari., Perez-Calvo, Estefania., Lopez-Ulibarri, Rual., Klausen, Mikkel. A muramidase from Acremonium alcalophilum hydrolyse peptidoglycan found in the gastrointestinal tract of broiler chickens. J. Ind. Microbiol. Biotechnol 2021;113–120. doi:10.1093/jimb/kuab008. PMC9113140 33693885

[47] Boroojeni FG, Männer K, Rieger J, Calvo EP, Zentek J. Evaluation of a microbial muramidase supplementation on growth performance, apparent ileal digestibility, and intestinal histology of broiler chickens. Poult. Sci. 2019;98(5):2080–2086. doi:10.3382/ps/pey556. 30566631

[48] Thaiss CA, Levy M, Grosheva I, Zheng D, Soffer E, Blacher E, Braverman S, Tengeler AC, Barak O, Elazar M, *et al*. Hyperglycemia drives intestinal barrier dysfunction and risk for enteric infection. Science 2018;359(6382):1376–1383. doi:10.1126/science.aar3318. 29519916

[49] Birchenough GMH, Nystrom EEL, Johansson MEV, Hansson GC. A sentinel goblet cell guards the colonic crypt by triggering Nlrp6-dependent Muc2 secretion. Science. 2016;352(6293):1535–1542. doi:10.1126/science.aaf7419. 27339979 PMC5148821

[50] Mar JS, Nagalingam NA, Song Y, Onizawa M, Lee JW, Lynch SV. Amelioration of DSS-induced murine colitis by VSL#3 supplementation is primarily associated with changes in ileal microbiota composition. Gut Microbes. 2014;5(4):494–503. doi:10.4161/gmic.32147. 25144681

[51] Hernández-Chirlaque C, Aranda CJ, Ocón B, Capitán-Cañadas F, Ortega-González M, Carrero JJ, Suárez MD, Zarzuelo A, Sánchez de Medina F, Martínez-Augustin O, *et al*. Germ-free and antibiotic-treated mice are highly susceptible to epithelial injury in DSS colitis. J. Crohn’s Colitis. 2016;10(11):1324–1335. doi:10.1093/ecco-jcc/jjw096. 27117829

[52] Li M, Wu Y, Hu Y, Zhao L, Zhang C. Initial gut microbiota structure affects sensitivity to DSS-induced colitis in a mouse model. Sci. China Life Sci. 2018;61(7):762–769. doi:10.1007/s11427-017-9097-0. 28842897

[53] Muniyappa R, Lee S, Chen H, Quon MJ. Current approaches for assessing insulin sensitivity and resistance in vivo: advantages, limitations, and appropriate usage. AJP Endocrinol. Metab 2007;294:E15–E26. doi:10.1152/ajpendo.00645.2007. 17957034

[54] Johansson, Malin E. V.Gustafsson, Jenny K.Sjöberg, Karolina E.Petersson, JoelHolm, LenaSjövall, HenrikHansson, Gunnar C. Bacteria penetrate the inner mucus layer before inflammation in the dextran sulfate colitis model. PLoS One. 2010;5(8):e12238. doi:10.1371/journal.pone.0012238. 20805871 PMC2923597

[55] Everard, Amandine., Belzer, Clara., Geurts, Lucie., Ouwerkerk, Janneke P., Druart, Céline., Bindels, Laure B., Guiot, Yves., Derrien, Muriel., Muccioli, Giulio G., Delzenne, Nathalie M., de Vos, Willem M., Cani, Patrice D. Cross-talk between Akkermansia muciniphila and intestinal epithelium controls diet-induced obesity. Proc. Natl. Acad. Sci. U. S. A. 2013;110(22):762–769. doi:10.1073/pnas.1219451110. PMC3670398 23671105

[56] Bian X, Wu W, Yang L, Lv L, Wang Q, Li Y, Ye J, Fang D, Wu J, Jiang X, *et al*. Administration of Akkermansia muciniphila ameliorates dextran sulfate sodium-induced ulcerative colitis in mice. Front. Microbiol. 2019;10:1–18. doi:10.3389/fmicb.2019.02259. 31632373 PMC6779789

[57] Håkansson Å, Tormo-Badia N, Baridi A, Xu J, Molin G, Hagslätt M-L, Karlsson C, Jeppsson B, Cilio CM, Ahrné S, *et al*. Immunological alteration and changes of gut microbiota after dextran sulfate sodium (DSS) administration in mice. Clin. Exp. Med. 2015;15(1):107–120. doi:10.1007/s10238-013-0270-5. 24414342 PMC4308640

[58] Reikvam, Dag Henrik., Derrien, MurielIslam, Rejoanoul., Erofeev, Alexander., Grcic, Vedrana., Sandvik, Anders., Gaustad, Peter., Meza-Zepeda, Leonardo A., Jahnsen, Frode L., Smidt, Hauke., Johansen, Finn-Eirik. Epithelial-microbial crosstalk in polymeric Ig receptor deficient mice. Eur. J. Immunol. 2012;42(11):2959–2970. doi:10.1002/eji.201242543. 22865203

[59] Berry D, Kuzyk O, Rauch I, Heider S, Schwab C, Hainzl E, Decker T, Müller M, Strobl B, Schleper C, *et al*. Intestinal microbiota signatures associated with inflammation history in mice experiencing recurring colitis. Front. Microbiol. 2015;6:1–11. doi:10.3389/fmicb.2015.01408. 26697002 PMC4678223

[60] Berry D, Schwab C, Milinovich G, Reichert J, Ben Mahfoudh K, Decker T, Engel M, Hai B, Hainzl E, Heider S, *et al*. Phylotype-level 16S rRNA analysis reveals new bacterial indicators of health state in acute murine colitis. ISME J. 2012;6(11):2091–2106. doi:10.1038/ismej.2012.39. 22572638 PMC3475367

[61] Yoshihara T, Oikawa Y, Kato T, Kessoku T, Kobayashi T, Kato S, Misawa N, Ashikari K, Fuyuki A, Ohkubo H, *et al*. The protective effect of bifidobacterium bifidum G9-1 against mucus degradation by Akkermansia muciniphila following small intestine injury caused by a proton pump inhibitor and aspirin. Gut Microbes. 2020;11(5):1385–1404. doi:10.1080/19490976.2020.1758290. 32515658 PMC7527075

[62] Malik A, Sharma D, Zhu Q, Karki R, Guy CS, Vogel P, Kanneganti T-D. IL-33 regulates the IgA-microbiota axis to restrain IL-1α-dependent colitis and tumorigenesis. J. Clin. Invest. 2016;126(12):4469–4481. doi:10.1172/JCI88625. 27775548 PMC5127671

[63] Nunberg M, *et al*. Interleukin 1α-deficient mice have an altered gut microbiota leading to protection from dextran sodium sulfate-induced colitis. mSystems 3. 2018 May 8;3(3):e00213-17.doi: 10.1128/mSystems.00213-17.eCollection May-Jun2018. 10.1128/mSystems.00213-17 PMC5940968 29766049

[64] Shono Y, Docampo MD, Peled JU, Perobelli SM, Velardi E, Tsai JJ, Slingerland AE, Smith OM, Young LF, Gupta J, *et al*. Increased GVHD-related mortality with broad-spectrum antibiotic use after allogeneic hematopoietic stem cell transplantation in human patients and mice. Sci. Transl. Med. 2016;8(339ra71):339ra71–339ra71. doi:10.1126/scitranslmed.aaf2311. PMC4991773 27194729

[65] Cani PD. Human gut microbiome: hopes, threats and promises. Gut. 2018:1–10. doi:10.1136/gutjnl-2018-316723. 29934437 PMC6109275

[66] Seregin SS, Golovchenko N, Schaf B, Chen J, Pudlo NA, Mitchell J, Baxter NT, Zhao L, Schloss PD, Martens EC, *et al*. NLRP6 Protects Il10 −/− Mice from colitis by limiting colonization of akkermansia muciniphila. Cell Rep. 2017;19(4):733–745. doi:10.1016/j.celrep.2017.03.080. 28445725 PMC5528001

[67] Plovier H, Everard A, Druart C, Depommier C, Van Hul M, Geurts L, Chilloux J, Ottman N, Duparc T, Lichtenstein L, *et al*. A purified membrane protein from Akkermansia muciniphila or the pasteurized bacterium improves metabolism in obese and diabetic mice. Nat. Med. 2017;23(1):107–113. doi:10.1038/nm.4236. 27892954

[68] Henke, Matthew T., Kenny, Douglas J., Cassilly, Chelsi D., Vlamakis, Hera., Xavier, Ramnik J., Clardy, Jon. Ruminococcus gnavus a member of the human gut microbiome associated with Crohn’s disease, produces an inflammatory polysaccharide. Proc. Natl. Acad. Sci. U. S. A. 2019;116(26):12672–12677. doi:10.1073/pnas.1904099116. 31182571 PMC6601261

[69] Ballal, Sonia A., Veiga, Patrick., Fenn, Kathrin., Michaud, Monia., Kim, Jason H., Gallini, Carey A., Glickman, Jonathan N., Quéré, G., Garault, P., Béal, C., *et al*. Host lysozyme-mediated lysis of Lactococcus lactis facilitates delivery of colitis-attenuating superoxide dismutase to inflamed colons. Proc. Natl. Acad. Sci. 2015;112(1):7803–7808. doi:10.1073/pnas.1501897112. 26056274 PMC4485081

[70] Sais M, Barroeta AC, López-Colom P, Nofrarías M, Majó N, Lopez-Ulibarri R, Pérez Calvo E, Martín-Orúe SM. Evaluation of dietary supplementation of a novel microbial muramidase on gastrointestinal functionality and growth performance in broiler chickens. Poult. Sci. 2020;99(1):235–245. doi:10.3382/ps/pez466. 32416807 PMC7587705

[71] Johansson MEV, Hansson GC. Preservation of Mucus in Histological Sections, Immunostaining of Mucins in Fixed Tissue, and Localization of Bacteria with FISH. Methods Mol. Biol. 2012:842;229–235. doi:10.1007/978-1-61779-513-8_13. 22259139

[72] Chassaing B, Koren O, Goodrich JK, Poole AC, Srinivasan S, Ley RE, Gewirtz AT. Dietary emulsifiers impact the mouse gut microbiota promoting colitis and metabolic syndrome. Nature. 2015;519(7541):92–96. doi:10.1038/nature14232. 25731162 PMC4910713

[73] Klindworth A, Pruesse E, Schweer T, Peplies J, Quast C, Horn M, Glöckner FO. Evaluation of general 16S ribosomal RNA gene PCR primers for classical and next-generation sequencing-based diversity studies. Nucleic Acids Res. 2013;41(1):1–11. doi:10.1093/nar/gks808. 22933715 PMC3592464

[74] Edgar R. UNOISE2: improved error-correction for Illumina 16S and ITS amplicon sequencing. bioRxiv. 2016:081257. doi:10.1101/081257.

[75] McMurdie PJ, Holmes S, Watson M. phyloseq: an R package for reproducible interactive analysis and graphics of microbiome census data. PLoS One. 2013;8(4):e61217. doi:10.1371/journal.pone.0061217. 23630581 PMC3632530

[76] Lozupone C, UniFrac KR. a New Phylogenetic Method for Comparing Microbial Communities. Appl. Environ. Microbiol. 2005;71(12):8228–8235. doi:10.1128/AEM.71.12.8228-8235.2005. 16332807 PMC1317376

[77] Oksanen, J., Blanchet, F. Guillaume., Kindt, Roeland., Legendre, P., Minchin, Peter R., O'Hara, R. B., Simpson, Gavin L., Solymos, Peter., Stevens, M., Henry H., Wagner, H. vegan: community Ecology Package. R Packag. Ver. 2017;2:3–4. doi:10.4135/9781412971874.n145.

[78] Love MI, Huber W, Anders S. Moderated estimation of fold change and dispersion for RNA-seq data with DESeq2. Genome Biol. 2014;15(12). doi:10.1186/s13059-014-0550-8. PMC4302049 25516281

[79] Douglas, Gavin M., Maffei, Vincent J., Zaneveld, Jesse R., Yurgel, Svetlana N., Brown, James R., Taylor, Christopher M., Huttenhower, Curtis,Langille, Morgan G. I. PICRUSt2 for prediction of metagenome functions. Nat. Biotechnol. 2020;38(6):685–688. doi:10.1038/s41587-020-0548-6. 32483366 PMC7365738

[80] Katakura K, Lee J, Rachmilewitz D, Li G, Eckmann L, Raz E. Toll-like receptor 9-induced type I IFN protects mice from experimental colitis. J. Clin. Invest. 2005;115(3):695–702. doi:10.1172/JCI22996. 15765149 PMC1051992

[81] Chassaing B, Srinivasan G, Delgado MA, Young AN, Gewirtz AT, Vijay-Kumar M. Fecal lipocalin 2, a sensitive and broadly dynamic non-invasive biomarker for intestinal inflammation. PLoS One. 2012;7(9):e44328. doi:10.1371/journal.pone.0044328. 22957064 PMC3434182

