# Supplementary material for: Fungal lysozyme leverages the gut microbiota to curb DSS-induced colitis
Source: Gut Microbes. 2021 Oct 25;13(1):1988836. doi: 10.1080/19490976.2021.1988836 (PMC8547870; doi:10.1080/19490976.2021.1988836)
Supplement: Supplemental Material [file KGMI_A_1988836_SM0756.docx]

**Figure S1: *In vivo* study designs**

**a)** Male C57BL6/J mice were fed high fat diet (HFD) or a source matched low fat reference diet (LFD) for 12 weeks while receiving daily oral gavage of 0.5 mg lysozyme or vehicle including n = 10 for LFD+Vehicle, n = 11 for HFD + Vehicle and HFD + Lyso groups. Body composition assessed by magnetic resonance (MR) scans and fecal microbiota composition by 16S rRNA gene amplicon sequencing was monitored every 4 weeks throughout the study period. At week 10 an oral glucose tolerance test (oGTT) with corresponding glucose-stimulated insulin secretion (GSIC) and a sulfonic acid assay of intestinal permeability were performed. The study ended with euthanasia and necropsy at 12 weeks where the small intestine microbiota was sampled. **b)** Female BALB/c mice were pre-treated vehicle or 0.02 or 1.0 mg lysozyme by oral gavage for two days (day -2 to 0) prior induction of colitis by DSS (day 0 to 5) with continuous daily lysozyme or vehicle administration until the day before euthanasia. Fecal microbiota was sampled at day -3 and day 0 before DSS was given. The study ended at day 5 with euthanasia and necropsy including microbiota sampling of ileum, cecum, and colon content. **c)** Similar study design as b, using exclusively the 1.0 mg lysozyme dose. The study included three groups receiving ampicillin and neomycin in drinking water from 5 days prior the start of lysozyme gavage. Mice were co-housed 4 by 4. **a-b)** Mice were co-housed 3 by 3.

**Figure S2: Body composition and intestinal gene expression and cytokines in HFD-fed C57BL6/J mice**

**a)** Fat mass in grams measured by magnetic resonance (MR) scans at the beginning and end of the study period. **b)** Lean mass in grams measured by MR scans at the beginning and end of the study period. **c)** Cumulated energy intake per week as average kcal per mouse in each cage throughout the study period. **d)** Glucose-stimulated insulin concentration (GSIC) during oGTT. Mixed-effects model with Geisser-Greenhouse correction and Dunnett’s multiple comparisons test to HFD+Vehicle group. **e)** Lipopolysaccharide (LPS) levels in plasma at the end of the study period. **f)** Fold-change in permeability markers *Occludin* and *Zonulin-1 (Zo-1)* gene expression of ileal tissue assessed by RT-qPCR. **g)** As f in colon tissue. **h)** Cytokine levels in colon tissue. **i)** Cytokine levels in ileum tissue.**a-b, e-i)** Graphs depict mean ± SEM with individual data points. **c-d)** Graph depicts group mean ± SEM. **a-i)** * = *p* < 0.05, ** *p* < 0.01, *** *p* < 0.001. Grey asterisk indicates significant difference between LFD+Vehicle vs HFD+Vehicle and black indicates HFD+Vehicle vs HFD+Lyso.

**Figure S3: Baseline gut microbiota and alpha diversity in HFD-fed C57BL6/J mice**

**a)** PCoA of baseline fecal microbiota (Week 0) using weighted UniFrac distance with each centroid indicating group mean. All groups were fed low fat reference diet at the sampled time point and later assigned the indicated experimental group. PERMANOVA test between LFD+Vehicle vs. HFD+Vehicle *p* = 0.76 and between HFD+Vehicle vs. HFD+Lyso *p* = 0.37. **d)** Observed species richness and Shannon’s diversity index of small intestine alpha diversity at the end of the study period sampled 3-5h after the last lysozyme/vehicle administration. **c)** Fecal alpha diversity measures Shannon’s Index and observed species richness (left) of fecal samples during the study sampled approximately 24h since the latest vehicle or lysozyme administration. Lines indicate group mean and 75% CI. **e)** Relative abundance in % of genera with differentially abundant (FDR corrected *p* < 0.05) ASVs in Fig 2e from baseline (0) over the study period (4-12) not shown in Fig 2e due to species dependent effects rather than whole genus changes. Graphs depict group mean and 75% CI. **f)** PCoA of KEGG orthologs using Bray-Curtis distances based on 16S rRNA gene amplicons of small intestine samples week 12. Centroids indicate the mean of each group. PERMANOVA test between LFD+Vehicle vs. HFD+Vehicle *p* = 0.45 and between HFD+Vehicle vs. HFD+Lyso *p* = 0.52.

**Figure S4: Lysozyme reproducibly and dose-dependently prevents Dextran-sulfate sodium (DSS)-induced colitis in BALB/c mice**

**a)** Body weight in grams as group mean ± SEM at baseline (day -3), at the start of DSS-challenge (day 0), and the following study period until end of the study (day 5). **b)** Body weight change from day 0 to 5 during as % of body weight. **c)** Colon damage assessed by Wallace histological scoring at the end of the study period. Bars indicate group median and interquartile range. Kruskal-Wallis test and Dunn’s multiple comparisons test to the DSS+Vehicle group. **d)** Colon length in cm**. e)** Interleukin **(**IL)-1β and IL-6 cytokine levels in colon tissue as fold change to vehicle group. **f)** IL-10 and IL-12 cytokine levels in colon tissue as fold change to vehicle group. **g)** IL-17A and IL-25 cytokine levels in colon tissue as fold change to vehicle group. **h)** TNFα cytokine levels in colon tissue as fold change to vehicle group. **i)** ASVs with differential abundance (FDR adjusted *p* < 0.05) by DEseq2 analysis comparing colon microbiota composition of DSS+Vehicle to Vehicle+Vehicle group at day 5 of the study sampled a day after the latest vehicle administration. ASVs are categorized with their classified genus and colored by their classified phylum. **j)** Mean relative abundance in % of 17 most abundant aggregated bacterial genera in ileum and cecum content samples of the end of the study. Missing entries indicate unclassified family and/or genus.

**k)** PCoA of KEGG orthologs using Bray-Curtis distances based on 16S rRNA gene amplicons of ileum and cecum samples week 12. Centroids indicate group means. **l)** Enzymatic activity of *A. alcalophilum*-derived lysozyme with or without Neomycin (Neo) and Ampicillin (Amp) measuring the fluorescent signal (excitation/emission of 485/520 nm) over time. The plot, which was representative for the data obtained from all tested lysozyme concentrations, depicts the results with a lysozyme concentration of 1 mg/mL selected as it mimicked the conditions tested in the *in vivo* experiments. **b,d-h)** Graphs depict mean ± SEM with individual data points. One-way ANOVA with Dunnet’s multiple comparisons test to DSS+Vehicle group. **a-h)** * = *p* < 0.05, ** *p* < 0.01, *** *p* < 0.001. Grey asterisk indicates significant difference between Vehicle+Vehicle vs DSS+Vehicle and black indicates comparisons between DSS+Vehicle vs Lyso-supplemented groups.
