# Supplementary material for: Fungal lysozyme leverages the gut microbiota to curb DSS-induced colitis
Source: Gut Microbes. 2021 Oct 25;13(1):1988836. doi: 10.1080/19490976.2021.1988836 (PMC8547870; doi:10.1080/19490976.2021.1988836)
Supplement: Supplemental Material [file KGMI_A_1988836_SM8837.docx]

**Supplementary Table 1: qPCR primer sequences and annealing temperatures**

| **Target** | **Forward Seq** | **Reverse seq** | **Annealing temp**  **Ileum** | **Annealing temp Colon** |
| --- | --- | --- | --- | --- |
| *Occludin* | ATGTCCGGCCGATGCTCTC | TTTGGCTGCTCTTGGGTCTGTAT | 61.5°C | 61.5°C |
| *Zo-1* | ACCCGAAACTGATGCTGTGGATAG | AAATGGCCGGGCAGAACTTGTGTA | 61.5°C | 61.5°C |
| *Tlr2* | GCAAACGCTGTTCTGCTCAG | AGGCGTCTCCCTCTATTGTATT | 62°C | 61.5°C |
| *Muc2* | CCCAGAAGGGACTGTGTATG | TTGTGTTCGCTCTTGGTCAG | 61.5°C | 61.5°C |
| *18S* | GTAACCCGTTGAACCCCATT | GTAACCCGTTGAACCCCATT | 62°C | 61.5°C |

**Supplementary Table *2:* Primer sequences for 16S rRNA gene amplification**

| **Universal bacteria primers (Illumina) used for C57BL6/J mice** |
| --- |
| **Illumina adapter and S-D-Bact-0341-b-S-17:**  5'-TCGTCGGCAGCGTCAGATGTGTATAAGAGACAGCCTACGGGNGGCWGCAG-3'  **Illumina adapter and S-D-Bact-0785-a-A-21:**  5'-GTCTCGTGGGCTCGGAGATGTGTATAAGAGACAGGACTACHVGGGTATCTAATCC-3'  **Universal bacteria primers used for BALB/c mice**  **341.2FDI:**   \| 5’-ACACTCTTTCCCTACACGACGCTCTTCCGATCTCCTACGGGNGGCWGCAG-3’ \| \| --- \|   **805.2RDI:**   \| 5’- AGACGTGTGCTCTTCCGATCTGACTACHVGGGTATCTAATCC-3’ \| \| --- \| |
